# Supplementary material for: Kisspeptin Is Upregulated at the Maternal-Fetal Interface of the Preeclamptic-like BPH/5 Mouse and Normalized after Synchronization of Sex Steroid Hormones
Source: Reprod Med (Basel). Author manuscript; Available in PMC 2023 Aug 3. (PMC10399610; doi:10.3390/reprodmed3040021)
Supplement: Supplementary Material [file NIHMS1915154-supplement-Supplementary_Material.pdf]

Article Supplement:

Kisspeptin Is Upregulated at the Maternal-Fetal Interface of the Preeclamptic-Like BPH/5 Mouse and Normalized after Synchronization of Sex Steroid Hormones

Viviane C. L. Gomes [1], Ashley K. Woods [2], Kassandra R. Crissman [1], Camille A. Landry [1], Kalie F. Beckers [1], Bryce M. Gilbert [3], Lucas R. Ferro [1], Chin-Chi Liu [1], Erin L. Oberhaus [3] and Jenny L. Sones [1,*]

Table S1. Forward and reverse *Mus musculus*-specific primer sequences used in qRT-PCR

Gene	Primer Sequence	Reference
18S	F: 5′GTAACCCGTTGAACCCCATT3′<br>R: 5′CCATCCAATCGGTAGTAGCG3′	Sones et al., 2016 [1]
Kiss1	F: 5' CGAAGGAGTTCCAGTTGTAGG3'<br>R: 5'AAGGAATCGCGGTATGCA3'	Zhang et al., 2014 [2]
Kiss1r	F: 5'CCGTCCAACGCTTCAGGAT3'<br>R: 5'GTGTAGCGAAAAACAGGGGAA3'	Zhang et al., 2014 [2]
Timp1	F: 5′ GACGGCCTTCTGCAATTCC3′<br>R: 5′ GTATAAGGTGGTCTGGTTGACTTCTG3′	Sakamuri et al., 2017 [3]
Timp2	F: 5′ GAGCCTGAACCACAGGTACCA3′<br>R: 5′ AGGAGATGTAGCACGGGATCA3′	Sakamuri et al., 2017 [3]
Timp4	F: 5′ CACCCTCAGCAGCACATCTG 3′<br>R: 5′ GGCCGGAACTACCTTCTCACT 3′	Sakamuri et al., 2017 [3]
Lif	F:5′TCAGCGACAAAGTTACTCCACCGT3′<br>R:5′AAGTGATGACAAAGCCCAACAGGC3′	Sones et al., 2016 [1]

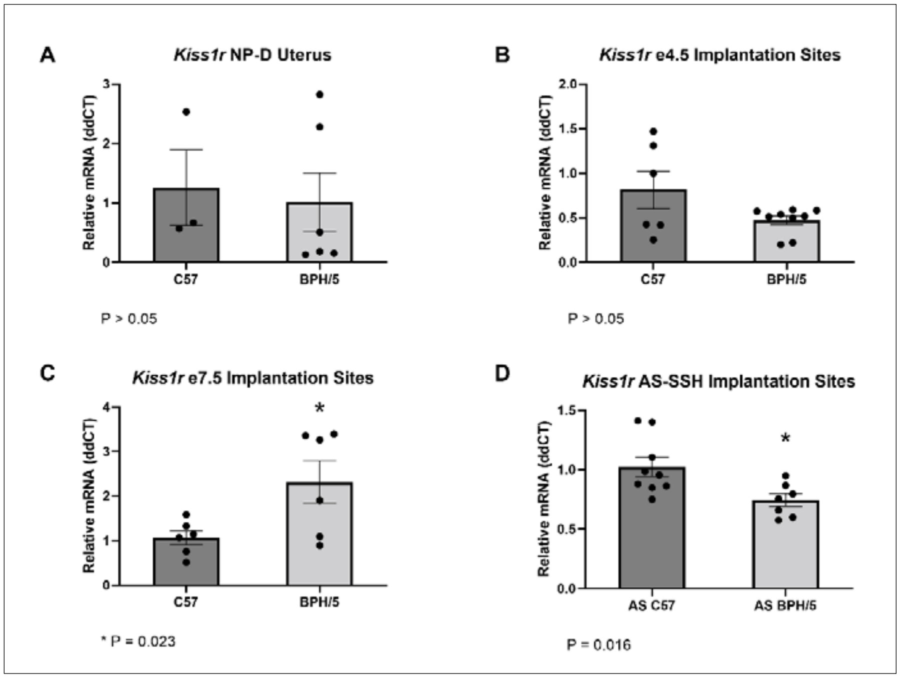

Supplement Figure S1. Kisspeptin receptor (*Kiss1r)* gene expression in the BPH/5 non-pregnant diestrus (NP-D) uterus and maternal-fetal interface. Relative mRNA expression of *Kiss1r* in the NP-D uterus (A), and embryonic implantation sites (eIS) of BPH/5 and C57 females carrying natural (NAT) pregnancies at embryonic day (e) 4.5 (B) and e7.5 (C). (D) *Kiss1r* expression during the peak of embryonic implantation in the eIS of BPH/5 and C57 females that underwent early pregnancy artificial synchronization of sex steroid hormones (AS-SSH). Gene expression assessed via qRT-PCR (n = 3-9/group). Student's t-test, * P < 0.05. Data expressed as mean ± SEM.

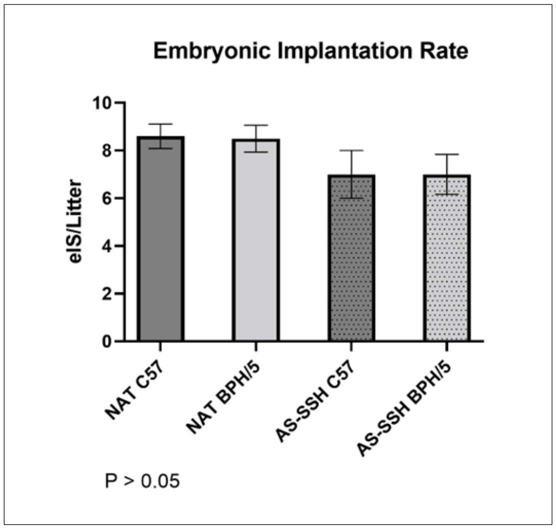

Supplement Figure S2. Embryonic implantation rate was not affected by artificial synchronization of sex steroid hormones (AS-SSH). Number of embryonic implantation sites (eIS) per litter in BPH/5 and C57 females carrying natural (NAT) pregnancies at embryonic day (e) 4.5, and BPH/5 and C57 females that underwent AS-SSH, two days post-administration of 17β-estradiol. One-way ANOVA, P > 0.05. Data expressed as mean ± SEM.
